# Recent Advances in the Microwave-Assisted Production of Hydroxymethylfurfural by Hydrolysis of Cellulose Derivatives—A Review

**DOI:** 10.3390/molecules23081973

**Published:** 2018-08-07

**Authors:** Frederic Delbecq, Christophe Len

**Affiliations:** 1 Ecole Superieure de Chimie Organique et Minerale, 60200 Compiegne, France; f.delbecq@escom.fr; 2 Universite de Technologie de Compiegne, Sorbonne Universites, 60200 Compiegne, France; 3 Chimie ParisTech, PSL University, 75005 Paris, France

**Keywords:** hydroxymethylfurfural, catalysis, microwave, green chemistry

## Abstract

The concepts of sustainable development, bioeconomy, and circular economy are being increasingly applied for the synthesis of molecules of industrial interest. Among these molecules, hydroxymethylfurfural as a platform molecule is the subject of various research approaches to improve its synthesis and productivity, and extend its potential uses. Accordingly, this review paper aims essentially at outlining recent breakthroughs obtained in the field of hydroxymethylfurfural production from sugars and polysaccharide feedstocks under microwave-assisted technology. The review discusses advances obtained via microwave activation in major production pathways recently explored, split into the following categories: (i) use of various homogeneous catalysts like mineral or organic acids, metal salts, or ionic liquids; (ii) feedstock dehydration making use of various solid acid catalysts; and (iii) non-catalytic routes.

## 1. Introduction

**Scheme 1:**
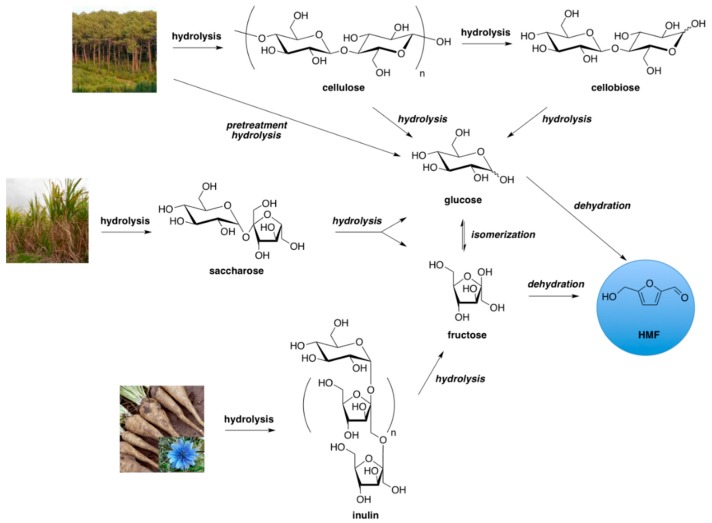
Main chemical pathways for 5-hydroxymethylfurfural (5-HMF) production from cellulose and carbohydrates.

**Scheme 2:**
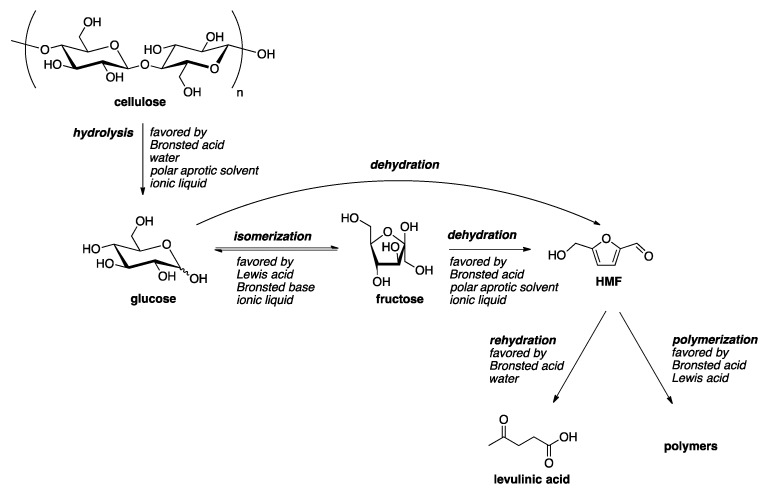
Chemical pathways for 5-HMF production from cellulose and carbohydrates.

Nowadays, from a global point of view, the need for basic functionalized carbon skeletons for fine chemical industry and the energy sector continues to grow day by day. However, our planet is facing an awareness of climate change, greenhouse gas emissions, and the rapid reduction of petroleum, increasing the price of fossil-fuel based materials. These concerns require the intensive production of biorenewable resources, including related residues through intensive processes into identified useful chemicals. The use of plant waste as recycled raw feedstocks is one of the alternatives for minimizing dependence on fossil oil. Chemical companies convert renewable bioresources into biofuels; platform molecules for fine chemicals; agro-chemicals; and specialty chemicals such as bio-lubricants, natural fibers, and bio-based solvents. Several building blocks derived from renewable resources, such as ethanol, glycerol, lactic acid, succinic acid, and levulinic acid, are already in use or considered with potential importance in the near future. Among them, furanic compounds, such as 2-furaldehyde (furfural) and 5-hydroxymethylfurfural (5-HMF) with various industrial applications [1,2,3,4,5,6,7,8,9,10,11,12,13,14,15], are conventionally manufactured from plant waste feedstocks via several steps. To date, many reports have shown that one-pot and efficient production of furans could be achieved from biomass by well-optimized catalysts, solvents, and equipment and process technology upgrades. Generally, 5-HMF was derived from cellulose or other hexose derivatives (e.g., saccharose) through a series of chemical processes: (i) hydrolysis of glucan to glucose; (ii) isomerization of glucose to fructose; and (iii) dehydration of fructose into 5-HMF. Starting from inulin (extracted from chicory) with only fructose as a building block, the process for the production of 5-HMF is favored even though the source is more expensive (Scheme 1). Among the different technologies used for the production of 5-HMF, acidic heterogeneous catalysis, acidic homogeneous catalysis, enzymatic catalysis, autocatalysis, and electrochemistry are the main processes. To date, different parameters are well known to enhance the 5-HMF yield and selectivity; among them, the nature of the catalyst (Bronsted acid, Lewis acid, Bronsted base), the acid strength, and the synergies between the catalytic sites and biphasic solvent systems to reduce the formation of side-products such as humins [16,17,18] (Scheme 2). However, it should be noted that very recently, dehydration of glucose to produce 5-HMF was done in the presence of Bronsted acid, bypassing the glucose–fructose isomerization [19]. An association of deep eutectic solvent, ionic liquids has also been studied.

**Scheme 3:**
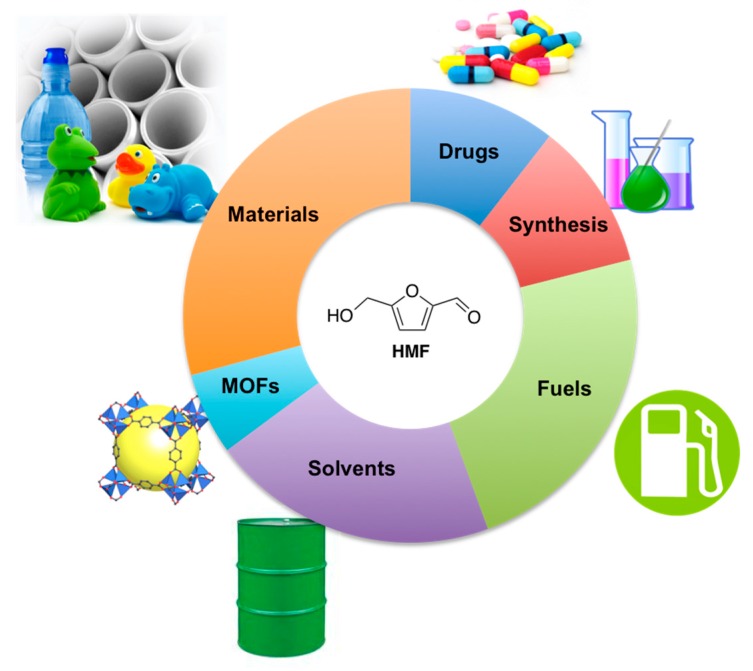
Main domains of practical applications of 5-HMF and its derivatives. MOFs—metal organic frameworks.

**Scheme 4:**
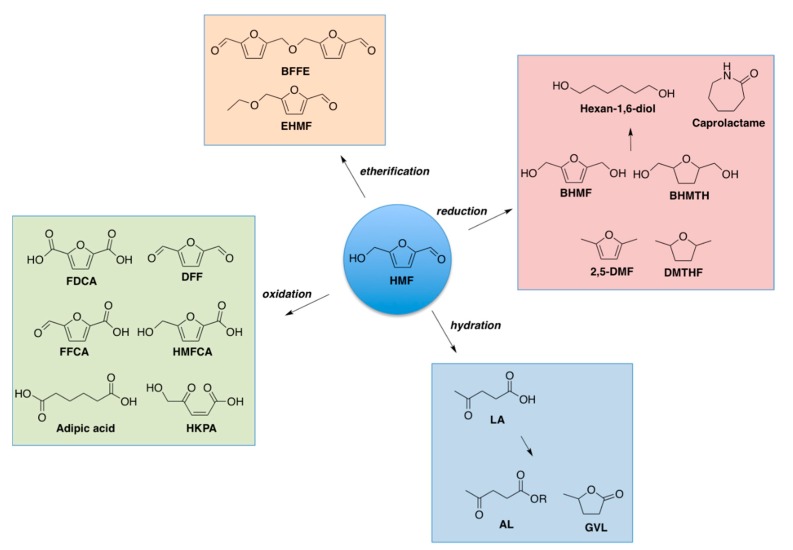
Catalytic transformation of 5-HMF into its derivatives.

As a molecule platform chemical, 5-HMF permits to produce a large range of chemicals with different properties and utilities as solvents, fuel additives, materials, and multiple fine chemicals (Scheme 3). The main pathways of 5-HMF reactivity are etherification, reduction, hydration, and oxidation (Scheme 4). 

Microwave (dielectric) radiation has been known for a long time, although its understanding and impact have evolved over time. In general, microwave ovens operate at wavelengths of either 12.2 cm (2.45 GHz) or 33.3 cm (900 MHz). In these conditions, microwave radiation creates energy-efficient internal homogeneous heating generated via electromagnetic field. This thermal effect permits to increase the rate of the reaction by a rapid rise in temperature due to (i) dipole rotation in the presence of a rapidly alternating electric field of microwave radiation and (ii) ionic conduction by rapid translational movements. Unlike conductive heating, the thermal mechanisms of rate acceleration are the superheating effect of solvents; the formation of so-called molecular radiators; the selective heating by using a strongly absorbing microwave catalyst or reagent in a less polar reaction medium; and the elimination of wall effects found in convective heating. A large number of green and sustainable approaches towards microwave-assisted organic synthesis (MAOS) have been reported, showing different advantages when compared with conventional heating protocols [20,21,22,23,24,25,26,27,28,29,30,31,32,33,34,35,36,37]. Microwave technology could also allow reactions at temperatures up to 300 °C and pressures up to 80 bar with safety priority and excellent parameter controls. To the best of our knowledge, microwave heating has only recently been developed for the production of 5-HMF [38]. This microwave activation is mainly carried out in a batch reactor even if few examples are reported using continuous flow devices. The purpose of the present article is to summarize the state-of-the-art in the field of 5-HMF production from sugars and polysaccharides using homogeneous and heterogeneous strategies under microwave activation in a batch process and in a continuous flow process. In general, the use of homogeneous catalysts gave very high selectivity and low conversion, while heterogeneous catalysts are very active and easy to separate, but not very selective.

## 2. Homogeneous Catalyst in Liquid

**Scheme 5:**
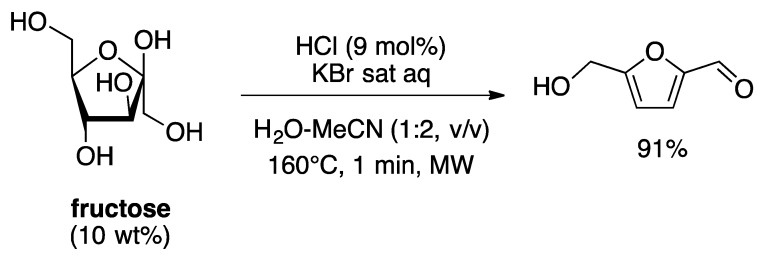
Catalytic transformation of fructose to 5-HMF using HCl in a biphasic system under microwave heating.

Mineral acids such as sulfuric or hydrochloric acid (H_2_SO_4_, HCl) were well studied for the production of furanic acids such as furfural or 5-HMF from the respective promoted dehydration of pentoses or hexoses substrates. For example, Repo and co-workers tried to determine the best conditions to transform fructose and other carbohydrates by successively changing the nature of the salt additive, often employed as a co-catalyst [39]. In the same work, variation of the nature of the acid and overall the solvent has been done. The optimized condition was first determined for fructose and the best acid was clearly HCl combined with potassium bromide (KBr) included in a water–acetonitrile mixture (1:2, *v*/*v*). When the solution was heated for 1 min at 160 °C, the conversion of fructose was complete with 91% of 5-HMF yield (Scheme 5). In the case of glucose, the yield was enhanced by addition of CrCl_3_·H_2_O, responsible for its possible isomerization into fructose prior to the usual dehydration process.

**Scheme 6:**
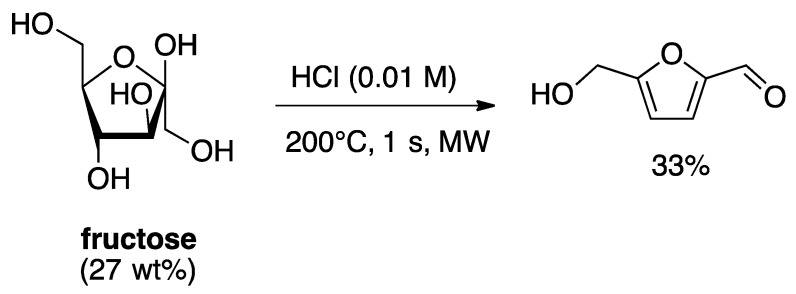
Catalytic transformation of fructose to 5-HMF using aqueous HCl under microwave heating.

Riisager and co-workers studied the influence of different parameters on the dehydration of fructose in HCl aqueous solution under microwave heating [40]. Successively, they measured the impact of temperature, pH, microwave power, and residence time on the yield of the formed 5-HMF. Finally, the higher 5-HMF value was obtained with a diluted fructose solution (27 wt %) prepared with 0.01 M HCl solution and the liquid was warmed up at 200 °C for 1 s. In this process, a fructose conversion of 52% and 5-HMF yield of 33% were achieved (Scheme 6). With a longer reaction time (60 s vs. 1 s), fructose conversion and 5-HMF yield were 95% and 53%, respectively.

**Scheme 7:**
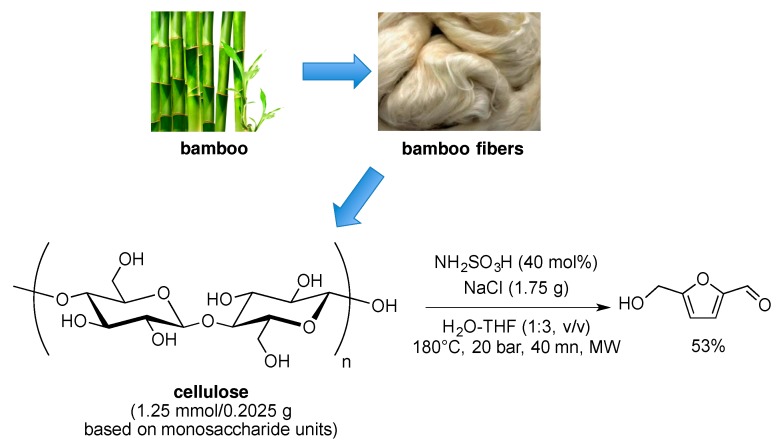
Production of 5-HMF from bamboo fibers hydrolysis catalyzed by NH_2_SO_3_H under microwave heating.

In the case of furfural, for converting pentoses such as xylose, organic acids are also reported to express remarkable ability for this purpose [41]. Wang and Sun developed a new methodology to transform the lignocellulosic biomass into 5-HMF [42]. In a H_2_O–THF (1:3, *v*/*v*) biphasic system, the authors were able to obtain 53% of 5-HMF yield by heating carbohydrates rich plant wastes such as the bamboo fibers in a microwave oven at 180 °C for 40 min (Scheme 7). The organic acid employed here was the sulfamic acid (NH_2_SO_3_H) in association with sodium chloride (NaCl), which could promote the reaction via stabilization of the carbocation intermediates and induce the phase transfer of the 5-HMF product. Using the same previous optimized condition, NH_2_SO_3_H also shows potential for dehydrating glucose into 5-HMF with 65% of yields.

**Scheme 8:**
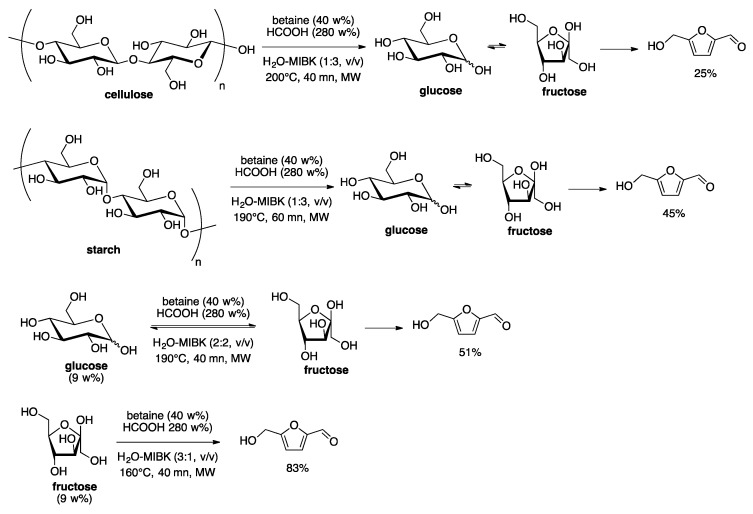
Production of 5-HMF from glucose, fructose, microcrystalline cellulose, and starch catalyzed by betaine–formic acid mixture under microwave heating.

As another recent example, Len and co-workers employed formic acid coupled with betain, a zwiterrionic small compound that could act as a co-catalyst for the dehydration of hexoses and glucose rich polysaccharides such as water soluble starch or microcristalline cellulose [38]. The reaction was performed in a water–methyl isobutyl ketone (MIBK) biphasic system of different volume ratios, and they measured their effects. All temperatures of each reaction were set up in a range found between 160 and 200 °C to get 5-HMF yields varying from 47 to 82% without trace of non desired levulinic acid (LA) (Scheme 8). The impact of substrate nature and catalyst amount in each case were also discussed in this work.

A similar strategy was developed by Xu and co-workers by direct transformation of microcristalline cellulose in dimethylacetamide (DMAc) containing a defined amount of LiCl (5 wt %) [43]. In fact, a small amount of an ionic liquid (IL) or 1,1,3,3-tetramethylguanidine tetrafluoroborate ([TMG] [BF4]) is employed as the catalyst in a lower concentration. Then, a maximum of 30% 5-HMF yield was obtained when 0.5 g of the cellulose was heated up under microwave heating at 120 °C for 2 h, even the optimized condition was originally defined for a solution of equal concentration in 100 mL of DMAc heated for 1 h at 140 °C. It should be noted that recent works described thermal and combustion risk profiles of ILs, as well as risks of toxicity and, consequently, a study of the stability and fate of the ILs in the process should be studied [44,45,46,47,48].

According to a recent paper by Tang and co-workers, a simple phosphate buffer system made of 0.83 M H_3_PO_4_ and 0.17 M NaH_2_PO_4_ was reported to be potent in producing 5-HMF from fructose or glucose, when each solution was heated up in a microwave oven at 150 °C for 30 min [49]. Only slight traces of LA were detected in the vessel and the yield of 5-HMF varied from 19 to 64%. Unfortunately, by increasing the charge of each carbohydrate, they observed the decrease of the 5-HMF yield, probably as a result of a large amount of undesired humin by-products generated during the process.

In 2018, Appels and co-workers reported the use of two batch reactors: one constructed of borosilicate glass and the other of silicon carbide (SiC) [50]. In the first one, the reaction mixture is heated via direct application of microwave radiation and, consequently, the heating is caused by dipole rotation and ionic conduction. In the second one, conventional wall heating is applied because SiC material protects the reaction mixture from the microwave radiation. Starting from cellulose using diluted HCl, the microwave heating using borosilicate glass vessel showed a positive influence on the rate of the hydrolysis of the polysaccharide (factor 2.3), and their employment was also expected to accelerate the isomerization of glucose into fructose (factor 2.5) for finally leading to the formation of the target 5-HMF. Using the SiC vessel, the yields of 5-HMF always remained inferior to those obtained with traditional glassware, and the complete transformation using SiC vessels was definitively more energy-consuming (factor 1.7). The difference between the two processes could be remedied by altering the stirring rates to avoid potential hot spots, as illustrated recently in another reaction model [51].

**Scheme 9:**
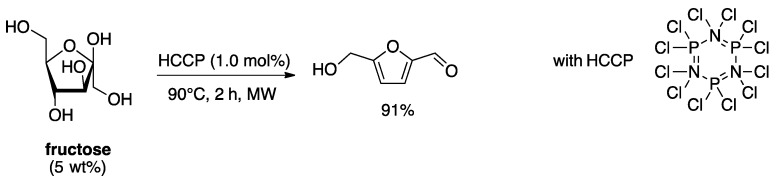
Catalytic transformation of fructose to 5-HMF using hexachlorocyclo triphosphazine (HCCP) under microwave heating.

Wang and Xu demonstrated the potential of chlorinated organic heterocycle compounds such as hexachlorocyclo triphosphazine (HCCP) and cyarunic chloride (CNC) as a homogeneous acid catalyst for the dehydration of fructose into 5-HMF in mild conditions [52]. These reactions were carried out in DMSO to avoid the formation of suspected by-products. The organic solution containing HCCP (1 mol %) was heated at 90 °C for 2 h to afford the furanic counterparts with a maximum of yield of 91% (Scheme 9). By replacing HCCP with CNC, 5-HMF was produced in 87% yield using a similar process. The authors explain that P–Cl and C–Cl bonds of HCCP and CNC have strong interactions between the ketose and the electron-withdrawing substituent promoting the dehydration. The risks of such catalysts should be especially considered during the dehydration process.

**Scheme 10:**
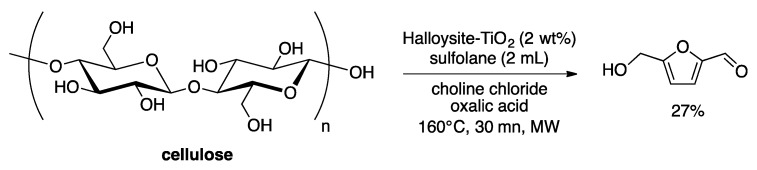
Catalytic cascade glucose transformation to 5-HMF using halloysite–TiO_2_ in deep eutectic solvent under microwave heating.

Another interesting study of Martin-Gil and co-workers described the capacity of various deep eutectic solvents considered as ionic liquids mixed with different catalysts to transform lignocellulosic wastes into both furfural and 5-HMF [53]. The most effective mixture was made of choline chloride and solid oxalic acid (5 mL), sulfolane (2 mL), water (2 mL) containing a small amount of halloysite as catalyst (2 wt %), and cellulose (100 mg). When heated at 140 °C for 30 min in a microwave oven, 5-HMF was produced in 27% yield (Scheme 10). The authors noted that ultrasonic pre-treatment (15 min) combined with stirring during the microwave heating represented the most favorable conditions.

**Scheme 11:**
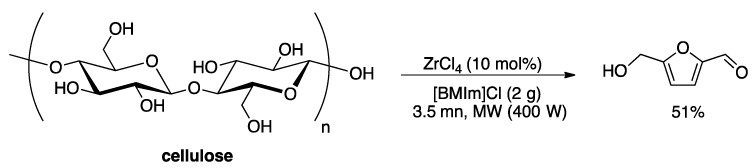
Catalytic transformation of cellulose to 5-HMF using ZrCl_4_ in [BMIm]Cl under microwave heating.

The use of Lewis acid such as ScCl_3_, ZrCl_4_, CrCl_3,_ AlCl_3_, and SnCl_4_ was reported. Even if Lewis acid favors the isomerization from glucose to fructose, interesting results were published even starting from polysaccharide and glucose. Using [BMIm]Cl as ionic liquid, Deng and co-workers reported the production of 5-HMF from different carbohydrates such as fructose, glucose, and sucrose with scandium (III) chloride (ScCl_3_) as catalyst [54]. When the solutions loaded with the sugars were heated at 400 W for 2 min under microwave radiation, the yields of 5-HMF from fructose, glucose, and sucrose were 95, 56, and 74%, respectively. Starting from sucrose, the recyclability was tested and catalytic dehydration was repeated up to six times using the same initial system filled with fresh sugar, but with a progressive 5-HMF yield reduction. In the same manner, 5-HMF was produced through the direct transformation of cellulose using zirconium (IV) chloride (ZrCl_4_) as catalyst also immersed in an ionic liquid [55]. Herein, successive hydrolysis of the hexose-rich polysaccharide and dehydration of the released free glucose sub-units were realized in the conventional [BMIm]Cl under microwave heating at 400 W. A short residence time of 3 min was enough to get 48% of 5-HMF yield from 100 mg of glucose in the presence of 10 mol% of the Lewis acid (Scheme 11). When the process was transposed to cellulose, using the optimized conditions, the yield of 5-HMF here reached a value of 51%. Keeping the same catalytic system unchanged, the reaction could be repeated for up to six cycles with just a slight diminishing of the 5-HMF yield.

Tsang and co-workers tested many trivalent and tetravalent metals chloride, such as stannyl (IV) chloride (SnCl_4_), to produce 5-HMF from starchy food wastes such as cooked rice [56]. For example, when heated in a range of temperature found between 120 and 160 °C for residence of time of 20 or 40 min, upon microwave radiation, the polysaccharides were easily hydrolyzed and the released glucose sub-units were effectively isomerized into fructose prior to the dehydration into 5-HMF. Starting from cooked rice, 23 wt % of 5-HMF yield was achieved after 40 min of reaction. Other types of food wastes could become the source of 13 wt % of 5-HMF yield on average.

The capacity of AlCl_3_ for the conversion of carbohydrates and polysaccharides into 5-HMF was studied. Rhagavan and co-workers employed a system involving aluminium chloride (AlCl_3_, 6 H_2_O) as a Lewis acid catalyst to perform the direct conversion of corn starch into 5-HMF [57]. This transformation was carried out in a commercial ionic liquid [BMIm]Cl. Even in the presence of a slight volume of water, they did not observe the rehydration of the furanic compound into levulinic acid (LA), because they used a binary system made of DMSO and [Bmim]Cl. This system was potent to obtain 5-HMF with 65% of maximum yield directly from waxy corn starch. Saha and co-workers preferred to use DMSO as the sole solvent and this nonprotic and polar liquid was compared with various miscible or not biphasic systems, such as water-MIBK, for the promotion of fructose, glucose, and sucrose dehydration [58]. Obviously, the best yields were obtained when DMSO was employed as the solvent, avoiding the formation of LA. In a general procedure, a mixture containing AlCl_3_ (50 mol %) was heated up at 140 °C for 5 min to dehydrate one of the above mentioned carbohydrates. Starting from fructose, glucose, and sucrose, yields of 5-HMF were 70%, 52%, and 42%, respectively. However, it appears more difficult to access to the desired free hexose sub-unit prior to dehydration by simply heating in the presence of the Lewis acid. Besides, both increase of the sugar loading and catalyst concentration have not had a beneficial impact on 5-HMF production. Interestingly, water soluble starch was reported to be a good substrate for 5-HMF production with 30% of the yield produced at the end of the process.

Zhao and co-workers investigated the possible dehydration of cellulose and glucose by always using CrCl_3_ as the catalyst in [MMIm]Cl ionic liquid for the production of 5-HMF via a microwave assisted transformation process [59]. When a solution containing 100 mg of glucose was heated at 400 W for 1 min in the presence of 3.6 wt % of the catalyst, the carbohydrate was converted in 5-HMF with 91% of yield. They also tried to transfer the procedure to the transformation of different types of glucose-rich polysaccharides and identified a cellulose giving 5-HMF yields of 62% as the best substrate for this objective. The good yields could be explained by the nature of the *O*-glycosidic bond between each glucose sub-unit of the polysaccharide backbone to be cleaved more easily by means of the catalyst. The same team extended the work and reported the potential of chromium (III) chloride (CrCl_3_) in 1-butyl-3-methylimidazole chloride ([BMIm]Cl) and bromide analogue ([BMIm]Br) to produce 5-HMF via the direct transformation of lignocellulosic wastes such pine wood or extracted cellulose [60]. Starting from cellulose and pine wood (100 mg) in the presence of CrCl_3_.6H_2_O (10 wt %) in [BMIm]Cl (2 g), microwave activation (400 W) during 2–3 min furnished 5-HMF in 62% and 52% yield, respectively. As usual, the use of pure compound (cellulose vs. pine wood) permitted having a better yield.

## 3. Heterogeneous Catalyst in Liquids

**Scheme 12:**
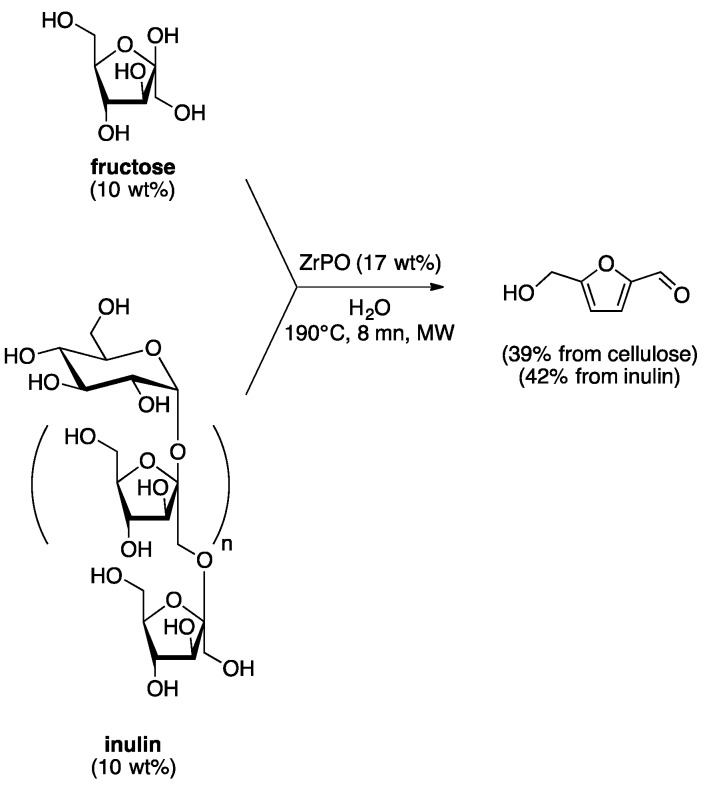
Catalytic transformation of fructose and inulin to 5-HMF using ZrPO in hot compressed water under microwave heating.

The use of supported and unsupported catalysts was studied for the production of 5-HMF [61]. Watanabe and co-workers tested sole titanium oxide (TiO_2_) and zirconium oxide (ZrO_2_) as catalysts for dehydration of both fructose and glucose into 5-HMF [62]. Usually, sugar (2 wt %) in water (5.0 mL) was heated at 200 °C for 1–10 min in the presence of 0.05 g of the catalyst. In case of fructose (2 wt %), the hot compressed water and selected heterogeneous catalysts TiO_2_ permitted to produce 5-HMF in 38% yield (conversion 84%) after 5 min of microwave heating. From glucose, 5-HMF could be also produced with 60% of yield and 50% of its conversion using ZrO_2_ as the catalyst in hot pressured water. The authors reported that ZrO_2_ acted as base and could promote the isomerization of glucose to fructose, whereas TiO_2_ could not only enhance the yield of 5-HMF, but also promote the isomerization step. Raspolli Galleti and co-workers employed niobium (NbPO) and zirconium phosphate (ZrPO) catalyst to dehydrate fructose and enulin in water under microwave heating without the addition of an extraction solvent [63]. NbPO and ZrPO present different acidic properties: amount of total acidic sites, Bronsted/Lewis acid ratio, and acidic strength. The optimal conditions were determined and fructose or inulin (10 wt %) aqueous solutions were heated at 190 °C for 8 min to give 41% and 43% of 5-HMF selectivity, respectively, in the presence of ZrPO as catalyst (Scheme 12). Interestingly, NbPO was found to be less active in the same conditions.

A similar study was realized using tin phosphate (SnPO) for dehydrating glucose into 5-HMF in ionic liquids [64]. The authors proved that SnPO is clearly able to generate 5-HMF with 58% of yield from glucose approaching 95% of the hexose conversion. Thus, the substrate needs to be heated in a microwave oven at 120 °C for 3 h in 1-ethyl-3-methylimidazolium bromide ([EMIm]Br). Currently, the mass ratio of catalyst–glucose was set up as 1:2 for all experiments.

Zhao and co-workers have carried out the conversion of fructose into 5-HMF in dimethyl sulfoxide (DMSO) using hybrid ZrO_2_ solid catalysts [65]. Initially, when a fructose solution is heated at 140 °C for 5 min in presence of ZrO_2_ and sulfate ions (SO_4_^2−^), the fructose conversion was quite complete for a 5-HMF yield of 73%. Later, some kind of modification of the material was realized by mixing ZrO_2_ with tungsten or molybdene oxides, referenced as WOx or MoOx, respectively. In fact, to express relative improved catalytic activity, the materials need to express good balance between Bronsted and Lewis acid properties. Then, when heated at 150 °C for 5 min, a 5 wt % fructose aqueous solution containing 10 wt % of SO_4_^2−^/WOx/ZrO_2_ or SO_4_^2−^/MoOx/ZrO_2_ catalysts can afford 83% and 82% of 5-HMF yield, respectively. In this work, the authors reported that SO_4_^2−^ is an electron-withdrawing group that could increase the formation of acidic centers after introduction into the material.

New macroporous polymer catalyst designed for use in high-temperature heterogeneous catalysis was tested for the 5-HMF production. When the solution containing the substrate is heated in the presence of the acidic resin at 180 °C for 20 min, 20 wt % of fructose aqueous solution or 10 wt % of inulin one gave 42% and 43% of the maximum of 5-HMF yield, respectively [66]. However, by increasing the sugar load, the catalyst could be deactivated by humin deposit on its surface, masking the sulfonic acid moieties. Using fructose as substrate, the catalyst remained active for up to seven cycles, but it needs to be regenerated by washing in acetone bath. It is also necessary to indicate that Repo and co-workers have also investigated the reaction promoted by Amberlyst 38 in the γ-valerolactone (GVL), a green solvent with a higher boiling point. Thus, fructose was transformed into 5-HMF with 74% of yield [39].

The use of polyvinyl alcohol (PVA) functionalized solid acid catalyst in low-boiling green solvent was studied for the 5-HMF production [67]. In this context, Pawar and Lali synthesized a new sulfated polyvinyl alcohol as a solid acid catalyst. The most promising solvent for this reaction was isopropyl alcohol, which represents the industrially most viable green solvent for the production of 5-HMF. In their hands, fructose (5.5 mmol) in the presence of DICA-T-1 (1.76 mg) in isopropanol (16 mL) was heated at 130 °C for 2 min to afford 5-HMF in 85% yield (conversion 95%). The maximum yield was obtained with a maximum of concentration of 6.25% of fructose.

Carbon nanotubes (CNTs) were tested as support for heterogeneous catalysis using microwave-assisted organic synthesis. The family of carbon materials such as CNTs, carbon black, graphene, and carbon fibers are ideal microwave absorbers because of their excellent dielectric polarization properties. In this context, the group of Zhu developed a new hybrid composite catalyst made of carbon nanotubes (CNTs) and TiO_2_ [68]. The fructose (0.55 mmol) dehydration was carried out in aqueous solution in the presence of 12.5 mg·mL^−1^ of the catalyst heated up under microwave radiation (15 W). When exposed for 30 min, 60% of 5-HMF yield was obtained. Furthermore, the thinner TiO_2_ layer is recommended to improve the intrinsic activity of the material. When performed in the same conditions, the reaction could afford 20% and 67% of 5-HMF yield from glucose and sucrose, respectively, under microwave heating for 40 min. The same group developed a new hybrid composite catalyst made of carbon nanotubes (CNTs) impregnated with polyaniline (PANI) [69]. By varying the amount of PANI on the surface of the CNTs, they created four different catalysts and one of them, composed of 30 wt % CNT, expressed the best performance to produce 5-HMF from fructose aqueous solution. The conversion of fructose was achieved after 30 min of heating with input power of 15 W in the presence of 12. 5 mg·mL^−1^ of the catalyst displaying 29 m^2^·g^−1^ of surface area. Herein, the catalytic effect is directly connected to the quality of the PANI coating that drives the energy transfer efficiency. This kind of catalyst could be efficiently reactivated by contact with H_2_SO_4_. In a normal procedure, by heating at 19 W for 20 min, it was sufficient to get 74% of 5-HMF yield.

## 4. Free-Catalyst in Liquid

Some researchers tried to prove that a specific solvent and superior temperatures are the sole conditions enough to produce 5-HMF in good yields from carbohydrates. First, conversion of fructose into 5-HMF without the use of an external catalyst in 1-butyl-3-methylimidazole chloride ([BMIm]Cl) was reported in 2011 [70]. A maximum of 98% of yield was recorded for a given volume of ionic liquid containing the carbohydrate was heated at 155 °C for 1 min. The conversion of the substrate was also close to 100%. The stability of the selected ionic liquid was not studied and partial degradation of it to furnish HCl could explain the published result.

Estrine and co-workers conducted dehydrations of various carbohydrates such as fructose, inulin, or sorbose into 5-HMF in DMSO itself as the sole solvent [71]. Thus, the polar aprotic organic solvent could act as promoter and stabilizer of the entire process, especially when the reaction is carried out at temperatures near or superior to 150 °C. The maximum of 5-HMF yield was obtained when the mixture of fructose (10 wt %) in DMSO was heated at 900 W for 4 min, with a remarkable value of 92% of selectivity (conversion 100%). With a fructose concentration superior to 10 wt %, a slight decrease of the 5-HMF selectivity was observed. Not surprisingly, the addition of a slight volume of water had a negative effect on the 5-HMF production during the process, especially when the reaction was carried out from inulin as the starting material.

## 5. Conclusions

This review summarizes the main catalytic processes under microwave activation for the biosourced synthesis of hydroxymethylfurfural. The nature of catalysis and catalysts were the guiding criteria taken into account. With respect to homogenous catalysis, Bronsted acid (e.g., HCl, NH_2_SO_3_H, HCOOH, H_3_PO_4_, and oxalic acid) and Lewis acid (e.g., ScCl_3_, ZrCl_4_, CrCl_3_, AlCl_3_, and SnCl_4_) were tested to produce 5-HMF from different starting materials. Even though it is well established that Bronsted acid favored the hydrolysis of polysaccharides and dehydration of fructose and Lewis acid favored the isomerization of glucose to fructose, the vast majority of researchers tested all the materials in case. In addition to the powerful homogeneous catalysis, the work developed by Len and co-workers deserves to be mentioned. Indeed, the use of formic acid coupled with betain opens new ways for homogeneous acidic catalysis. However, the corrosive, environmental, and handling problems should be appropriately taken into consideration with homogeneous acid catalysts. Concerning heterogeneous catalysis, metal oxide (e.g., TiO_2_, ZrO_2_), alone or coupled (e.g., SO_4_^2−^/WOx/ZrO_2_ or SO_4_^2−^/MoOx/ZrO_2_), furnished 5-HMF in excellent yield. Supported acid polymers (e.g., Amberlyst-70, PVA) and CNT were also active in the production of 5-HMF. Solid catalysts lie in their complicated, and therefore relatively costly, synthesis process. Despite these drawbacks, some of them do have inherent properties that might deserve valuable applications for the biorefinery concept. Two reports showed that 5-HMF can be obtained without acid catalysts; one with the use of [BMIm]Cl and the potential formation of HCl, and the second with the use of harsh conditions and the potential formation of HCOOH. The use of ILs permited to produce 5-HMF with excellent yields, but a major drawback pertains to their separation difficulty from the chemicals formed when used in biorefining, which still today act as a genuine barrier for their industrial use.

Application of microwave activation is showing great promise for the production of 5-HMF from biomass. This opens a more cost-effective process that might meet the target for scale-up. As microwave chemistry around 5-HMF is still a mostly uncharged territory, the chemists have to pave the way in this area.

## Abbreviations

The following abbreviations are used in this manuscript:

AL	alkyl levulinate
BFFE	bis (5-formyfurfuryl) ether
BHMF	2,5-bis (hydroxymethyl) furan
BHMTHF	2,5-bis (hydroxymethyl) tetrahydrofuran
[BMIm]Br	1-butyl-3-methylimidazolium bromide
[BMIm]Cl	1-butyl-3-methylimidazolium chloride
CNC	cyanuric chloride
CNT	carbon nanotube
DFF	2,5-diformylfuran
DICA-T-1	acronyme from the authors
DMAc	dimethylacetamide
2,5-DMF	2,5-dimethylfuran
DMSO	dimethyl sulfoxide
DMTHF	2,5-dimethyltetrahydrofuran
EHMF	5-ethoxymethylfurfural
[EMIm]Br	1-ethyl-3-methylimidazolium bromide
FDCA	2,5-furandicarboxylic acid
FFCA	5-formylfuran-2-carboxylic acid
GVL	γ-valerolactone
HCCP	hexachlorocyclo triphosphazine
HKPA	5-hydroxy-4-keto-pent-3-enoic acid
5-HMF	5-hydroxymethylfurfural
5-HMFCA	5-(hydroxymethyl)furan-2-carboxylic acid
IL	ionic liquid
LA	levulinic acid
MAOS	microwave-assisted organic synthesis
[MMIm]Cl	1-methyl-3-methylimidazolium chloride
MIBK	methyl isobutyl ketone
MOF	metal organic framework
NbPO	niobium phosphate
PANI	polyaniline
PVA	polyvinyl alcohol
SiC	silicon carbide
SnPO	tin phosphate
TMG	tetramethylguanidine
ZrPO	zirconium phosphate
